# Testing the gearbox hypothesis for insect flight control

**DOI:** 10.1242/jeb.250768

**Published:** 2026-10-16

**Authors:** Abin Ghosh, Girish Kumar G.S., Sanjay P. Sane

**Affiliations:** National Centre for Biological Sciences, Tata Institute of Fundamental Research, GKVK Campus, Bellary Road, Bengaluru 560065, India

**Keywords:** Radial stop, Pleural wing process, Wing hinge, Yaw maneuvers, *Musca domestica*

## Abstract

Across Diptera, sustained flight relies on stretch-activated asynchronous flight muscles that generate multiple contraction cycles per motor neuron impulse, enabling wingbeat frequencies of ∼100–1000 Hz. These muscles attach to the thorax as indirect flight muscles, driving wing flapping through thoracic deformation, while smaller steering muscles modulate wing kinematics stroke by stroke. The wings articulate with the thorax through a wing hinge, where the radial stop (RS) and grooved pleural wing process (PWP) are proposed to act as a mechanical ‘gearbox’ that modulates wingbeat amplitude during maneuvering. Supporting this hypothesis, previous studies observed that close RS–PWP interactions correlated with discrete wingbeat amplitude changes during fictive turns in tethered flies, although the functional significance of this in freely flying insects remained untested. Here, we first examined the morphological diversity of the wing hinge across Diptera using scanning electron microscopy and found substantial variation in PWP morphology, suggesting that this putative gearbox is not highly conserved. We next tested gearbox function by bilaterally ablating the PWP in freely flying houseflies (*Musca domestica*), released into an L-shaped arena requiring 90 deg yaw turns. Kinematic analyses revealed no significant differences between control and PWP-ablated flies in wing or body kinematics during yaw turns. Both groups differentially modulated wingbeat amplitude to generate yaw torques, indicating that PWP is inessential for wingbeat amplitude modulation and that active neuromuscular control, rather than passive RS–PWP engagement, predominates in regulating wingbeat amplitude during maneuvers. Our findings challenge a central prediction of the gearbox hypothesis and highlight the robustness of the dipteran flight control system.

## Teaser

**Highlighted Article:** This study tests the gearbox hypothesis, which was proposed to explain how flies control their wing hinge to modulate aerial turns. We show that this gearbox model may be incomplete or incorrect, because ablating the components of the wing hinge does not alter turning performance in free flight.

## INTRODUCTION

The evolution of flight enabled insects to expand their ecological niches, contributing to their morphological and physiological diversification (Grimaldi and Engel, 2005; Treidel et al., 2024). Insects vary in size over three orders of magnitude, from a few hundred micrometers in fairy fly wasps (*Kikiki huna*, Hymenoptera) (Polilov, 2012) to wingspans of 300 mm in large saturniid moths (*Attacus* sp., Lepidoptera) (Dudley, 2000; Sane, 2016). To generate sufficient lift, smaller insects must flap their wings at higher frequencies (Ellington, 1984; Deora et al., 2017). Whereas the common housefly *Musca domestica* flaps its wings at more than 200 Hz, in certain midges (*Forcipomyia* sp., Diptera), wingbeat frequencies can approach 1000 Hz (Sotavalta, 1953). In these insects, control of wing motion must operate at similarly rapid rates, making them an ideal system in which to probe the mechanisms of rapid neural processing and motor output.

How do thoracic muscles achieve such high frequencies? Skeletal muscles exhibit an intrinsic trade-off between force production and shortening velocity arising from the kinetics of actin–myosin cross-bridge cycling (Josephson, 1985; Syme and Josephson, 2002). In synchronous muscles, where each contraction is triggered by neural input, contraction frequency is constrained by neural stimulation rate and the activation–relaxation kinetics of calcium. However, in many insects, including Diptera, Hymenoptera and Coleoptera, these constraints are overcome by the presence of asynchronous flight muscles, in which delayed stretch activation and thoracic coupling generate self-sustained oscillations, enabling high wingbeat frequencies through phase-lagged force production (Pringle, 1949; Josephson et al., 2000; Deora et al., 2017). In these muscles, contraction frequency is decoupled from motor neuron firing and cycle-by-cycle calcium transients, with neuronal inputs maintaining the levels of calcium while contraction cycles are mechanically triggered via delayed stretch activation of antagonistic muscle sets. The interaction between stretch-activated muscle dynamics and thoracic elasticity sustains thorax–wing oscillations, allowing multiple contraction cycles per neural activation and enabling the high frequencies and power output required for flight (Gau et al., 2023).

Within the thorax, these asynchronous muscles are organized in the indirect flight muscle (IFM) architecture: antagonistic dorso-ventral muscles (DVMs) and dorso-longitudinal muscles (DLMs) attach to the inner side of the exoskeleton and deform it during contraction. DVM contraction compresses the thorax dorsoventrally and elongates it longitudinally, activating DLMs after a delay, whereas DLM contraction produces the opposite deformation and activates DVMs. This reciprocal mechanical coupling sustains cyclic deformation without one-to-one neural activation (Pringle, 1949; Dickinson and Tu, 1997; Deora et al., 2017). The underlying motor neurons fire at rates far below wingbeat frequency, serving primarily to maintain intracellular calcium and modulate power output via changes in IFM spike rate (Gordon and Dickinson, 2006; Lehmann et al., 2013; Lehmann and Bartussek, 2017).

Because the IFMs connect to the thoracic walls instead of the wing base, a separate mechanism is required to transform thoracic deformations into coordinated wing upstroke and downstroke. This transmission is mediated by the wing hinge, a complex assembly of sclerites actuated by steering muscles. Because of their asynchronous nature, IFMs cannot provide stroke-to-stroke control. Instead, fine modulation of wing kinematics during maneuvers is achieved by synchronous direct flight muscles, or steering muscles, which contract once per neural spike, allowing precise, cycle-specific control. These smaller muscles have a well-developed sarcoplasmic reticulum and fast-twitch kinetics (Dickinson et al., 1998; Lehmann and Bartussek, 2017). In the flesh fly *Sarcophaga dux*, 19 such muscles coordinate the motion of each wing, 14 of which insert directly on the sclerites (Deora et al., 2017). While IFMs generate the primary power for wing oscillation, steering muscles control nuanced changes in wing attitude. The synchronization of left and right wing phase is mediated by the scutellum, a mechanical linkage in the thorax (Deora et al., 2015, 2017). This linkage, along with a weaker linkage connecting each wing to its ipsilateral haltere, ensures wing–wing synchrony during flight and fixed haltere phase relative to the wings. Thus, the wings and halteres move as dual-coupled oscillators, in which wing–wing coordination is strongly coupled, whereas wing–haltere coordination is weakly coupled (Deora et al., 2015, 2021). Within these fixed phase relationships, precise wing movement is mediated by the wing hinge, which is finely controlled by the underlying steering muscles (Melis et al., 2024).

To study the control of flight, it is essential to investigate both steering muscles and the mechanics of the sclerites at the wing base, which together form the wing hinge. The wing hinge functions as a system of interconnected levers, enabling muscle forces to translate into precise wing movements during flight and other behaviors such as courtship (e.g. Bennet-Clark and Ewing, 1968; Shirangi et al., 2013). Whereas significant progress has been made in elucidating the combinatorial roles of steering muscles in flies and their effects on wing kinematics (Nachtigall and Wilson, 1967; Wisser and Nachtigall, 1984; Tu and Dickinson, 1994, 1996; Trimarchi and Schneiderman, 1995; Fayyazuddin and Dickinson, 1996; Heide and Götz, 1996; Balint and Dickinson, 2001; Gordon and Dickinson, 2006; Walker et al., 2014; Sadaf et al., 2015; Lehmann and Bartussek, 2017; Lindsay et al., 2017; Melis et al., 2024), the mechanical contribution of the wing hinge remains comparatively less understood.

Noticing that the large flesh fly *Sarcophaga bullata*, under CCl_4_ anesthesia, held its wings either in fully upstroke or fully downstroke positions, Boettiger and Furshpan (1952) proposed a ‘click’ mechanism in Diptera, arguing that the thoracic structure was bistable. This meant that the wings adopted one of two stable states at the extreme ends of the wing stroke, enabling rapid transitions between wing strokes. This view was challenged by Miyan and Ewing (1985a,b), who argued that, under CCl_4_ exposure, the ‘click’ was an artefact of excessive tension in the pleuro-sternal muscles, which span the pleural and sternal regions of the thorax and contribute to deformation and stiffening of the thoracic exoskeleton. Based on tethered-flight observations of *M. domestica*, *Drosophila melanogaster* and *Drosophila virilis*, they proposed that the pleural wing process (PWP), a thoracic process arising from the dorsal end of the pleural ridge and projecting into the wing hinge, acts as a stopping point and secondary pivot during the downstroke. The PWP interacts with the radial stop (RS), a proximal process of the radial wing vein, and failure of this interaction was proposed to cause abnormal wing movements. Based on functional morphological observations of the wing hinge in *Calliphora erythrocephala*, Pfau (1987) argued that the RS interacts with the two grooves of the PWP as part of the dipteran click mechanism. Ennos (1987) observed RS engagement primarily with the anterior groove of the PWP in *Calliphora*, and hypothesized that posterior groove interaction generates asymmetric wingbeat amplitudes for turning. In tethered-flight observations of *Lucilia sericata*, *Sarcophaga argyrostoma* and *Glossina morsitans*, Miyan and Ewing (1988) found instances where the RS did not engage the PWP, and showed that removing the RS led to ipsilateral loss of amplitude control and wing jitter. Wisser (1988) proposed that in *Calliphora erythrocephala*, there are three RS contact points on the PWP instead of two, attributing downstroke amplitude variation to these interactions and suggesting that the PWP is primarily used in extreme maneuvers to avoid structural wear. In addition to the RS–PWP system, traditional descriptions of the wing hinge include a third component, pterale C, thought to function as a mechanosensory structure providing wingbeat frequency feedback or as a damper cushioning the impact of the wing at ventral stroke reversal (Ritter, 1911). Together, the RS–PWP action and the function of pterale C may be termed the ‘gearbox’ hypothesis, following Nalbach (1989), who conducted extensive kinematic studies on tethered *C. erythrocephala*.

Nalbach (1989) tested the gearbox hypothesis in *C. erythrocephala* using synchronized stroboscopic videography to capture both macroscopic wing motion and microscopic hinge mechanics during tethered flight. She identified three distinct downstroke modes based on whether and where the RS contacted the PWP. These modes correlated with discrete wingbeat amplitudes and were asymmetrically employed during spontaneous turning maneuvers, providing experimental evidence that hinge mechanics could contribute to flight control by modulating stroke amplitude. In studies that examined steering muscle activity during wing flapping in *Calliphora vicina*, Balint and Dickinson (2001) made similar observations about ‘mode switching’, which they argued was controlled by the muscles of axillary sclerites I and III. In *S. dux*, Deora et al. (2015) observed PWP mode shifts during flight initiation, transitioning from posterior to anterior grooves with increasing amplitude, before the RS eventually moved anterior to the PWP without interaction (Fig. 1A). In the hoverflies *Eristalis tenax* and *Eristalis pertinax*, Walker et al. (2012) showed that a hinged flap at the wing base, the alula, switches state mid-downstroke, coinciding with RS–PWP engagement and changes in stroke amplitude, deviation and supination timing. This flipped state was associated with reduced aerodynamic force and altered acceleration, suggesting that it marks shifts in wing hinge configuration and flight mode.

Across these studies, the PWP emerges as a crucial structure influencing wingbeat amplitude modulation and maneuverability through its interactions with the RS. However, these studies relied on observations of the wing hinge in tethered flies and correlations with kinematic parameters to infer gearbox function. Moreover, although these studies were typically carried out on muscomorphic flies, the importance of fine-amplitude modulation extends across flies, and perhaps other insects. Hence, a comparative exploration of gearbox structure is essential to understand the wing hinge and its function in flight. It is also essential to carry out these studies in freely flying insects, in which multiple turning strategies may be employed in addition to gearbox function.

The gearbox hypothesis is predicated on the assumption that the RS–PWP interaction is crucial for amplitude modulation. It is also possible that wing amplitude is primarily modulated by steering-muscle control of the sclerites. These hypotheses are not mutually exclusive, as both the wing hinge and steering muscles are essential components of the wing transmission system that converts thoracic vibrations into wing movement. Testing them requires us to address several questions. First, how variable is gearbox morphology across Diptera? Second, how do the relative configurations of the RS and PWP influence wing and body movements during turns? Third, how does selective modification of the wing hinge compromise insect flight? To address these questions, we investigated the structural and functional role of the wing hinge in flight control, focusing on the RS–PWP interaction in freely flying houseflies, *M. domestica*. We conducted an electron microscopic examination of the wing hinge, focusing on PWP, RS and pterale C morphology across several dipteran taxa to determine structural variability. We next investigated the functional role of the RS–PWP system in modulating wingbeat amplitude by ablating the PWP and testing the flies' ability to perform controlled yaw turns. Together, these studies challenge the validity of the gearbox hypothesis and indicate that resolving its role in flight requires a more detailed understanding of how steering muscles modulate wing hinge configuration.

## MATERIALS AND METHODS

### Scanning electron microscopy (SEM) of the wing hinge

Wild dipteran insects were collected opportunistically from the campus of NCBS, Bangalore, using soft insect nets. After collection, they were fixed in 4% paraformaldehyde (PFA) to preserve the structural integrity of their thorax and associated structures. Wings were positioned in the fully elevated position during fixation to facilitate imaging of the wing hinge. Fixed specimens underwent serial dehydration in ethanol grades of 20%, 30%, 50%, 60%, 75%, 90%, and twice in 100%, for 10 min each. To prevent morphological distortion during drying, the ethanol-dehydrated samples were subjected to critical point drying using a Leica EM CPD 300 Critical Point Dryer (Leica Microsystems, Wetzlar, Germany) for 90 min. Dried samples were mounted on aluminium stubs using conductive carbon adhesive tape. A gold coating (∼10 nm) was applied using an Emitech K550X sputter coater (Quorum Technologies Ltd, Laughton, East Sussex, UK) for 5 min to enhance conductivity. Imaging was performed using a Zeiss Merlin Compact VP SEM at an accelerating voltage of 2–5 kV and a working distance of 10–15 mm using the SE2 detector.

**Fig. 1.**
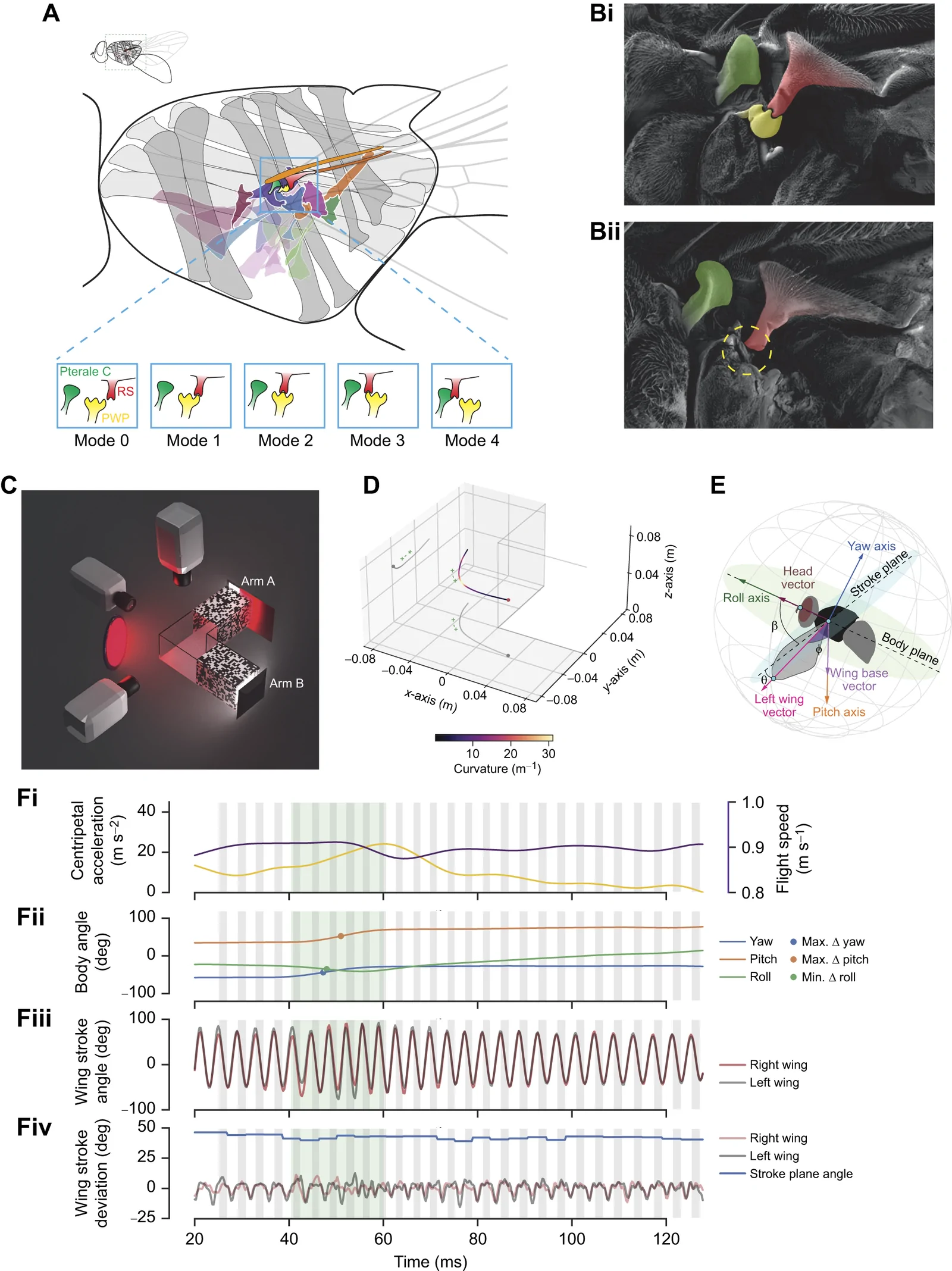
**Methodology to investigate the effect of pleural wing process ablation on flies executing a yaw turn.** (A) Schematic diagram of the fly wing hinge and proposed interaction modes between the radial stop (RS, red) and pleural wing process (PWP, yellow) in *Musca domestica*. Pterale C is shown in green, the scutellar lever arm in orange and the parascutal shelf (PSS) with pleurosternal muscles in violet. Major wing base sclerites and associated steering muscles are illustrated: axillary muscle 1 (AX1, solid blue; associated muscles, translucent blue), basalar sclerite and muscles B1–B3 (wine red), axillary sclerite 3 and muscles III1–III4 (magenta), and axillary sclerite 4 and muscles Hg1–Hg4 (green). The indirect flight muscles, dorso-ventral muscles (DVMs) and dorso-longitudinal muscles (DLMs), are shown in grey. Blue insets show the proposed RS–PWP interaction modes. In mode 0, the RS lies posterior to the PWP without contact; in modes 1–3, the groove on the RS sequentially engages the three PWP peaks; and in mode 4, the RS moves anterior to the PWP without contact. These hinge elements form part of the exoskeletal linkage system that transmits thoracic deformation to wing motion in Diptera. (B) Scanning electron micrographs of the wing hinge in *M. domestica* showing (i) intact PWP and (ii) ablated PWP. The PWP was mechanically ablated using microsurgical scissors. The PWP is highlighted in yellow, the RS in red and pterale C in green. The dashed yellow circle marks the ablation site after PWP removal. (C) The L-shaped flight arena used to induce left turns in *M. domestica*. Flies initiated flight from arm A toward arm B in an L-shaped box covered with a high-contrast random checkerboard pattern for visual guidance. Three high-speed cameras, placed approximately orthogonal to each other, captured top, front and side views at the junction for 3D reconstruction. High-intensity tungsten lighting filtered through a deep red filter provided illumination for high-speed recording while minimizing behavioral interference. White LED panels at the ends of arms A and B acted as visual attractants, and two additional white LED panels above the checkered walls (not shown) increased ambient light and supported sustained flight. Once the flies settled in arm A, a red filter was slid across the backlight, inducing a startle response through a sudden perceived decrease in light intensity and causing flies to fly toward the junction and execute a left yaw turn into arm B. (D) Center-of-mass (COM) trajectory of a flying *M. domestica*, color coded by local curvature computed using the Frenet–Serret framework. The green dashed line marks the trajectory segment used for comparative analysis. (E) Fly-centric coordinate system used to compute body and wing kinematics. The head vector extends from the midpoint of the wing bases to the head, and the wing base vector joins the right and left wing bases. Roll, pitch and yaw were computed about the head vector axis, wing base axis and axis normal to the head and wing base vectors, respectively. The head and wing base vectors define the body plane. The left wing vector joins the left wing base and tip. The average stroke plane for each wingbeat was estimated by least-squares regression of wing tip trajectories. The angle between the body plane and stroke plane defines the stroke plane angle (β). In the stroke plane-aligned frame, stroke angle (φ) describes in-plane wing motion and stroke deviation (θ) describes out-of-plane motion. (F) Overview of flight parameters. (i) Centripetal acceleration (yellow) and speed (violet) of the fly COM. (ii) Body angle: yaw (green), pitch (orange) and roll (blue), with dots marking the points of maximum rate of change for each rotational axis. (iii) Left (dark gray) and right (red) wing stroke angle. (iv) Left (dark gray) and right (pink) wing stroke deviation, together with stroke plane angle (sky blue). The green shaded region marks the segment selected for analysis; gray shaded regions indicate downstrokes.

### Animal maintenance for free flight experiments

Adult *Musca domestica* Linnaeus 1758 specimens were collected from wild populations and housed in ventilated containers with *ad libitum* food and water using cotton pads soaked in a 10% sugar solution and fresh water, at 25°C. Specimens were used within 2 days of collection to minimize potential effects of prolonged laboratory acclimation on their physiological state.

### PWP ablation

Male *M. domestica* specimens with no wing damage, mites or other visible deformities were selected from the housing container for PWP ablation. Flies were cold anesthetized in a 50 ml centrifuge tube kept in ice for 1 min. To maintain anesthesia during the ablation procedure, each specimen was positioned on a brass block maintained on ice. To expose the PWP, the wings were held in the upstroke position and the PWP was bilaterally ablated using fine microscissors (15,000-08, Fine Science Tools, Foster City, CA, USA). The ablation procedure, including anesthetization, was completed within 5 min to prevent any adverse effects of prolonged exposure to ice. Flies were allowed to recover in a 50 ml centrifuge tube overnight with *ad libitum* water but no sugar. Any specimens exhibiting unusual wing resting positions after overnight recovery were excluded from further experimentation to ensure consistency in the data. Control specimens underwent the same anesthetization protocol but did not undergo PWP ablation. PWP ablation was verified after the experiment using a dissection microscope to ensure the integrity of the ablation procedure (Fig. 1Bii).

### Free-flight experimental setup to induce left turns in houseflies

Free-flight experiments were conducted in an L-shaped flight box designed to induce a left turn during flight, starting from arm A towards arm B (outer arm length 36 cm, inner arm length 27 cm, cross-section 9 cm×9 cm; Fig. 1C). The arms of the box, except at the junction, were covered with a high-contrast black-and-white random checkerboard pattern to provide visual cues for navigation. Three high-speed cameras, v1212 (capturing at 1280×800 pixel resolution), VEO 640L (1152×1100 pixel resolution) and v611 (1280×800 pixel resolution) (Phantom, Vision Research Inc.), were set up roughly orthogonal to each other at the junction to capture top, front and side views, enabling 3D reconstruction of flight trajectories. The cameras operated at 4000 frames s^−1^ with an exposure time of 70 µs to obtain a crisp image of their wing outlines. This ensured that each wing stroke was captured within approximately 20 frames. The flight box was illuminated from the top using a white LED panel to provide adequate lighting for flight (∼3500 lx). A bright tungsten lamp covered with a red filter provided additional illumination optimized for the cameras without interfering with the flies' responses to visual stimuli. At the back end of arm A, a white LED panel was used to trigger a startle response in the flies. When a red filter was suddenly slid in front of this light, the flies exhibited a reflexive startle response and flew toward the junction of the L-box. Upon reaching the junction, the flies turned left and proceeded toward the white light at the end of arm B (Fig. 1C).

### Calibration

The flight box was securely mounted on an opto-mechanical breadboard equipped with multiple guide bolts to maintain a consistent position relative to the cameras throughout the experiments. Calibration of the three camera views (top, front and side) was performed based on easyWand (Theriault et al., 2014). A thin capillary of 75 mm length, suspended by a thread, was moved around the junction of the L-box in various orientations and positions. Approximately 50–70 point pairs, digitized from the ends of the capillary, were used to estimate the 11 parameters of the direct linear transformation (DLT) matrix, which maps 3D real-world coordinates to their corresponding 2D projections in the three camera views. The 3D coordinate system was defined with the lower inner corner of the L-box at the junction as the origin. Arm A of the box was aligned with the *y*-axis, arm B was aligned with the *x*-axis, and the height of the box was aligned with the *z*-axis. The DLT coefficients were updated to add the rotational and translational components to meet the above criteria. This coordinate system provided a consistent frame of reference for analyzing flight kinematics across trials.

### Digitization of the video and analysis

To quantify the flight kinematics, six key anatomical points – the head, left wing base, left wing tip, right wing base, right wing tip and abdomen tip – were manually annotated for each camera view using DLTdv8 (Hedrick, 2008). The 3D coordinates of these points were calculated using the DLT calibration coefficients derived from the wand calibration method. For body kinematic analysis, the 3D coordinates were low-pass filtered using a Butterworth filter with a cut-off frequency of 100 Hz to remove high-frequency oscillations from wing oscillations close to 200 Hz while preserving body motion characteristics. For wing kinematics analysis, the coordinate points were low-pass filtered using a Butterworth filter with a cut-off frequency of 1000 Hz to remove high-frequency noise.

### Flight speed and curvature of the flight trajectory

The center of mass (COM) of the fly was estimated as the midpoint between the left and right wing bases. The velocity vector (**v**) was derived as the first temporal derivative of COM position. To calculate the curvature κ of the fly's COM trajectory in 3D, we used the Frenet–Serret frame. The Frenet–Serret frame describes the local geometry of a curve in space using three mutually orthogonal unit vectors: the tangent **T** (direction of motion), the normal **N** (direction of turning) and the binormal **B** (orthogonal to both).

The rate of change of the tangent vector **T** with respect to arc length *s* is directed along the normal vector **N** and scaled by the curvature κ:

(1) $$\frac{dT}{ds}=\kappa N.$$

We used a custom MATLAB function, which computed the Frenet–Serret frame of a 3D curve. Curvature was computed from the three-dimensional center of mass trajectory recorded as time series data in *x*, *y* and *z* coordinates. The first step involved estimating the velocity vector at each time point by numerically differentiating the position data using finite differences. The speed, or arc length differential, was then calculated as the magnitude of the velocity vector. This speed was used to normalize the velocity and obtain the unit tangent vector, which describes the direction of motion along the trajectory. Next, the rate of change of the tangent vector with respect to time was computed, and this derivative was rescaled using the arc length to obtain the change of tangent vector with respect to distance traveled along the curve. Curvature was finally calculated as the magnitude of this arc length-based derivative of the tangent vector (Fig. 1D).

### Calculation of dynamic body angle

Dynamic changes in yaw, pitch and roll were computed iteratively for each frame based on the head vector (red line joining the COM to the head point; Fig. 1E) and wing base vectors (purple line passing through the left and right wing bases; Fig. 1E). Initial values of yaw, pitch and roll with respect to the global coordinate system were determined using the head and wing base vectors from the first frame. Yaw and pitch were obtained by converting the head vector into spherical coordinates, while roll was calculated from the transformed base vector.

For each subsequent frame, the local coordinate system was established using the following basis vectors: the *z*-axis as the unit vector of the cross-product of the head and base vectors, the *x*-axis as the normalized head vector, and the *y*-axis as the cross-product of the *z*-axis and *x*-axis vectors. The transformed base vector was then rotated to align with the yaw and pitch adjustments, and roll was computed from its spherical coordinates. The head and base vectors of the current frame were transformed into the defined local coordinate system. Changes in yaw and pitch were then calculated by converting the transformed head vector into spherical coordinates. The cumulative sums of Δ yaw, Δ pitch and Δ roll were added to their respective initial values to compute the overall yaw, pitch and roll for each frame. Because turning dynamics are more directly reflected in angular velocity than in absolute body orientation, rates of change of yaw, pitch and roll were used for statistical comparison rather than the absolute body angles themselves.

### Calculation of wing kinematic parameters

Wing kinematics were calculated in MATLAB from the three-dimensional coordinates of the left and right wing bases and wing tips in each video frame. For each frame, the left wing vector (magenta line joining the left wing base and left wing tip; Fig. 1E) and a right wing vector were defined from wing base to wing tip, and these vectors were transformed into a body-centered coordinate system using the body yaw, pitch and roll estimated from the head position and the line joining the two wing bases. The body frame wing vectors were then used to segment the motion into individual wingbeats from the extrema of the filtered stroke angle trace.

For each wingbeat, the stroke plane was estimated from the pooled left and right wing tip trajectories in the body frame by fitting a least-squares plane (sky-blue plane; Fig. 1E). The normal vector to this fitted plane was normalized and constrained to have a positive *z*-component, and its orientation was converted to a stroke plane angle. This procedure yielded one representative stroke plane per wingbeat, thereby preserving wingbeat-to-wingbeat variation in stroke plane orientation.

The body frame wing vectors were then rotated by the estimated stroke plane angle about the pitch axis to align the mean stroke plane with the analysis frame. In this stroke plane-aligned frame, wing orientation was expressed in spherical coordinates. Stroke angle was calculated from the azimuth of the rotated wing vector relative to a lateral reference axis through the wing bases, with the right wing sign inverted so that left and right wings followed the same sign convention. Thus, stroke angle represented the in-plane angular excursion of the wing within the stroke plane (purple arc, angle φ; Fig. 1E). Stroke deviation (or deviation from mean stroke plane; Sane and Dickinson, 2001) was defined as the elevation of the wing vector in the stroke plane-aligned frame and therefore quantified the out-of-plane displacement of the wing relative to the mean stroke trajectory (θ; Fig. 1E). Fig. 1F shows a sample trajectory with the extracted values of centripetal acceleration (Fig. 1Fi), body angle (Fig. 1Fii), wing stroke angle (Fig. 1Fiii) and wing stroke deviation (Fig. 1Fiv) as functions of time.

Wingbeat amplitude was quantified separately for the left and right wings from the stroke angle trace, and trial-wise minimum, maximum and median wingbeat amplitudes were extracted within the turn window for statistical comparison. Wingbeat amplitude difference was calculated as the difference between right and left wingbeat amplitude and summarized in the same way. Stroke deviation was defined as the elevation of the wing vector in the stroke plane-aligned frame. For comparison across trials, stroke deviation traces were segmented into left and right downstrokes and upstrokes using the detected stroke boundaries, and for each segment class we quantified both the maximum absolute stroke deviation and the median of the segment-wise maximum absolute stroke deviation within the turn window. These trial-wise summary metrics were then used for group-level statistical analysis.

### Trajectory selection and comparison

To ensure consistency across trajectories and experimental groups, we first identified the time point corresponding to the peak centripetal acceleration. From this point, a subsection of 80 consecutive frames was extracted for all trajectories and used as the basis for comparison. Restricting analysis to this standardized temporal window reduced variability caused by spontaneous fluctuations in flight behavior. At the acquisition rate of 4000 frames s^−1^, this interval corresponds to 0.02 s of flight. Given the median wingbeat frequency of the flies in our experiments (∼267 Hz), this duration encompasses approximately five wingbeat cycles, providing a representative yet controlled segment of kinematic data for analysis. This window length lies within the time scale over which rapid flight maneuvers in flies occur, where key changes in body dynamics and aerodynamic torque are produced over only a few wingbeats and complete turning maneuvers typically occur within roughly 10 wingbeats (Fry et al., 2003; Muijres et al., 2014).

### Statistical analysis

A total of 10 insects were analyzed per treatment. Although each insect underwent multiple flight trials, only one trial per insect was digitized and included in the analyses. To avoid pseudo-replication and the associated risk of Type 1 errors, all statistical tests were performed at the trial level. For each trial, a turn window was defined as the 81 frames preceding (and including) the selected best turn location, identified from the heading-change peak and its associated centripetal acceleration peak, and a single summary value for each plotted variable was extracted from this window. These variables included curvature, centripetal acceleration, speed, angular rates of yaw, pitch and roll, wingbeat frequency, stroke plane angle, wingbeat amplitude asymmetry and the stroke deviation summary metrics used in the box plots. Trials were grouped as control or PWP ablated.

For the standard two-group box plots, normality was assessed with the Shapiro–Wilk test within each group, and group comparisons were performed using two-sided Mann–Whitney *U*-tests, to determine whether there was any significant difference between two independent, non-normally distributed groups. For wing-specific wingbeat amplitude plots, left versus right comparisons within a group were tested using paired Wilcoxon signed-rank tests (which is a non-parametric test used to compare two related, matched or repeated-measures samples when the data do not follow a normal distribution). Between-group comparisons for a given wing were performed using Mann–Whitney *U*-tests. The stroke deviation box plots were analyzed in the same way as the other grouped comparisons, with separate two-sided Mann–Whitney *U*-tests performed for each plotted deviation metric.

## RESULTS

### The grooved gearbox morphology is not conserved across dipterans

The gearbox hypothesis proposes that the RS–PWP interaction is crucial for amplitude modulation, suggesting that its structure should be conserved across Diptera. We compared the RS, PWP and pterale C structures in the wing hinge of 10 specimens sampled across the dipteran phylogenetic tree (Fig. 2A–J): *Pselliophora* spp., *Clogmia* spp., *Tabanus* spp., *Hermetia illucens*, *Condylostylus* spp., *Allobaccha* spp., *Drosophila melanogaster*, *Musca domestica*, *Sarcophaga dux* and *Calliphora* spp. The RS was identified as the end structure of the radial vein at the wing base, and the PWP as the protrusion near the wing base arising from the pleural wall (Figs 1B, 2A–J). Pterale C, when present, was identifiable as the third component of the wing hinge, arising from the thoracic wall anterior to the PWP. In the dipteran insects sampled here, pterale C was absent in three groups, including Psychodomorpha (drain flies, *Clogmia* spp.; Fig. 2B), Tabanomorpha (horseflies, *Tabanus* spp.; Fig. 2C) and Empidoidea (long-legged flies, *Condylostylus* spp.; Fig. 2E), which are not monophyletic. This suggests that pterale C may be a conserved feature of Diptera that was secondarily lost in these members.

**Fig. 2.**
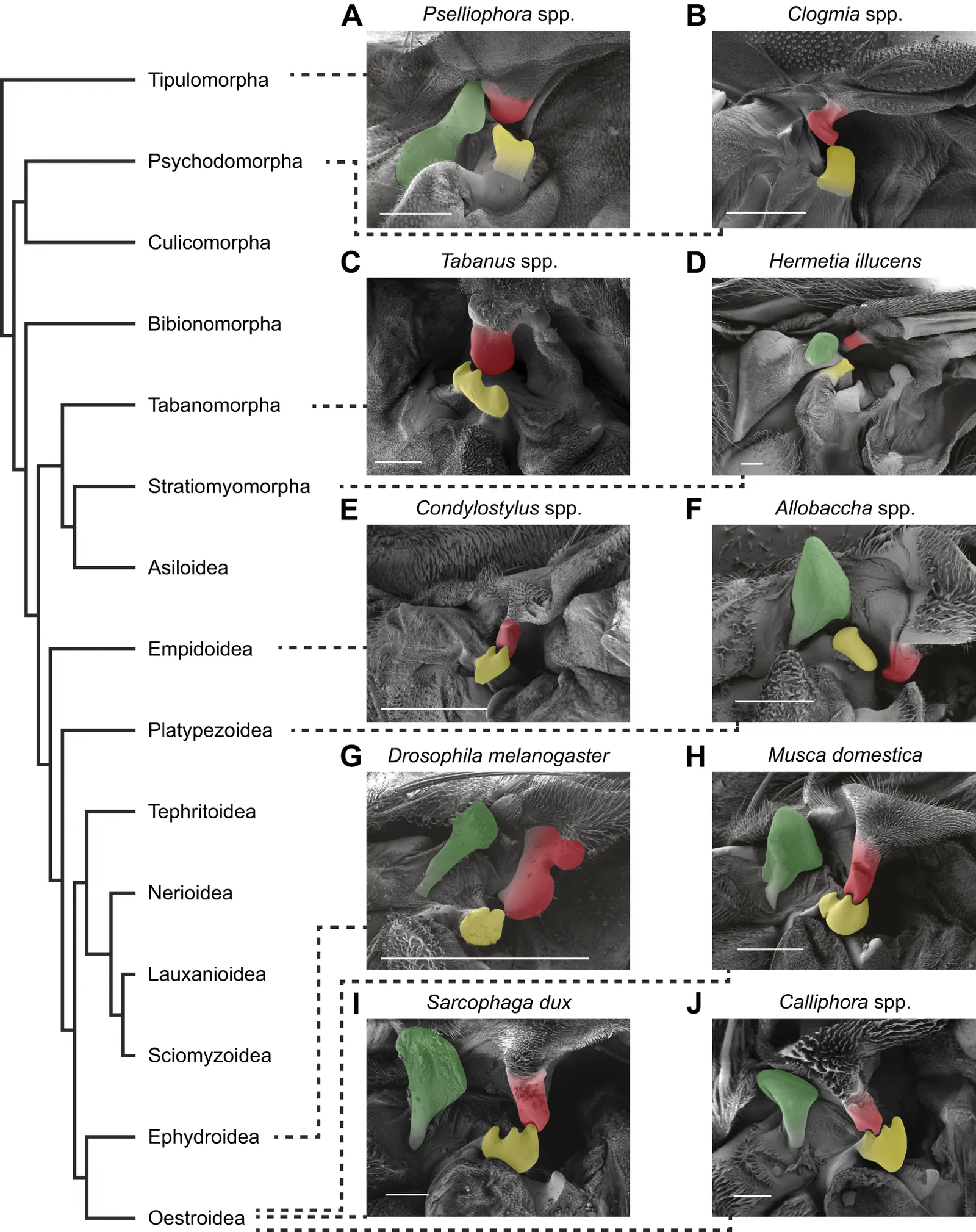
**Morphological diversity of the wing hinge in 10 dipteran insects.** (A–J) Scanning electron micrographs of the wing bases of representative species, sampled from across the dipteran phylogeny. Pterale C is false-colored in green, the PWP in yellow, and the RS in red. Species were selected to capture morphological variation across major dipteran lineages, which included Tipulomorpha (*Pselliophora* spp.; A), Psychodomorpha (*Clogmia* spp.; B), Tabanomorpha (*Tabanus* spp.; C), Stratiomyomorpha (*Hermetia illucens*; D), Empidoidea (*Condylostylus* spp.; E), Platypezoidea (*Allobaccha* spp.; F), Ephydroidea (*Drosophila melanogaster*; G) and three Oestroidea (*Musca domestica*, H; *Sarcophaga dux*, I; and *Calliphora* spp., J)*.* The phylogenetic relationships among the sampled species are shown alongside (phylogenetic tree reproduced from Wiegmann et al., 2011). Scale bars: 100 μm.

The RS and PWP were present and identifiable across all 10 specimens. However, their morphology is variable and does not show an obvious correspondence within the sampled taxonomic groups. For example, in the groups sampled here, the PWP is ungrooved in Tipulomorpha (Fig. 2A), Psychodomorpha (Fig. 2B), Stratiomyomorpha (Fig. 2D) and Platypezoidea (Fig. 2F). In Empidoidea and Ephydroidea (Fig. 2E,G), the PWP contains a single groove, whereas in Tabanomorpha and Oestroidea, it is multi-grooved (Fig. 2C,H–J).

These findings demonstrate that the two-grooved morphology of the PWP is not a conserved feature across Diptera, indicating structural divergence. Although detailed and standardized flight performance measurements are not available for these groups, there appears to be no clear correlation between flight ability and wing hinge structure. For example, horseflies (Tabanomorpha) fly at high speeds and over long distances (Wilkerson and Butler, 1984) but lack pterale C. Similarly, hoverflies (Platypezoidea) are extremely maneuverable and require precise modulation of wing amplitude (Walker et al., 2010), but their PWP is ungrooved. In Tabanomorpha and Oestroidea, the RS interdigitates with PWP grooves, suggesting that their shapes may have evolved to interact and minimize slippage. Together, these data suggest that gearbox morphology is highly variable and does not clearly correlate with flight ability, as would be predicted by the gearbox hypothesis.

### Both control and PWP-ablated flies performed the yaw turn through a banked maneuver

How important is the PWP for generating free-flight turns? According to the gearbox hypothesis, wingbeat amplitude modulation during aerial turns requires interaction of the RS and PWP. Hence, we analyzed free-flight kinematics of houseflies performing leftward turns in an L-shaped flight chamber. Across 20 flies, including 10 control individuals and 10 flies with bilaterally ablated PWP, all animals executed leftward turns and showed similar overall trajectories (Fig. 3A). These maneuvers were consistent with a banked turn: flies first rolled to the left, reflected by a negative roll angle and a tilt of the dorsal side toward the inner side of the turn (Fig. 3Bi), then pitched up (Fig. 3Bii) and yawed left through the turn (Fig. 3Biii). The maneuver therefore consisted of three phases: an initial roll phase, a pitch-up phase and a counter-roll phase that returned the animal to a dorsal-side-up orientation after the turn (Fig. 1E; Movie 1). Of the 20 flies tested, 18 clearly exhibited all three phases. In the remaining two flies, the dorsal side was already oriented toward the inner side of the turn before entering the calibrated volume, and the initial roll phase was therefore not observed. Together, these observations indicate that houseflies use a structured banked-turn strategy during leftward turning, and that this pattern is preserved after PWP ablation.

**Fig. 3.**
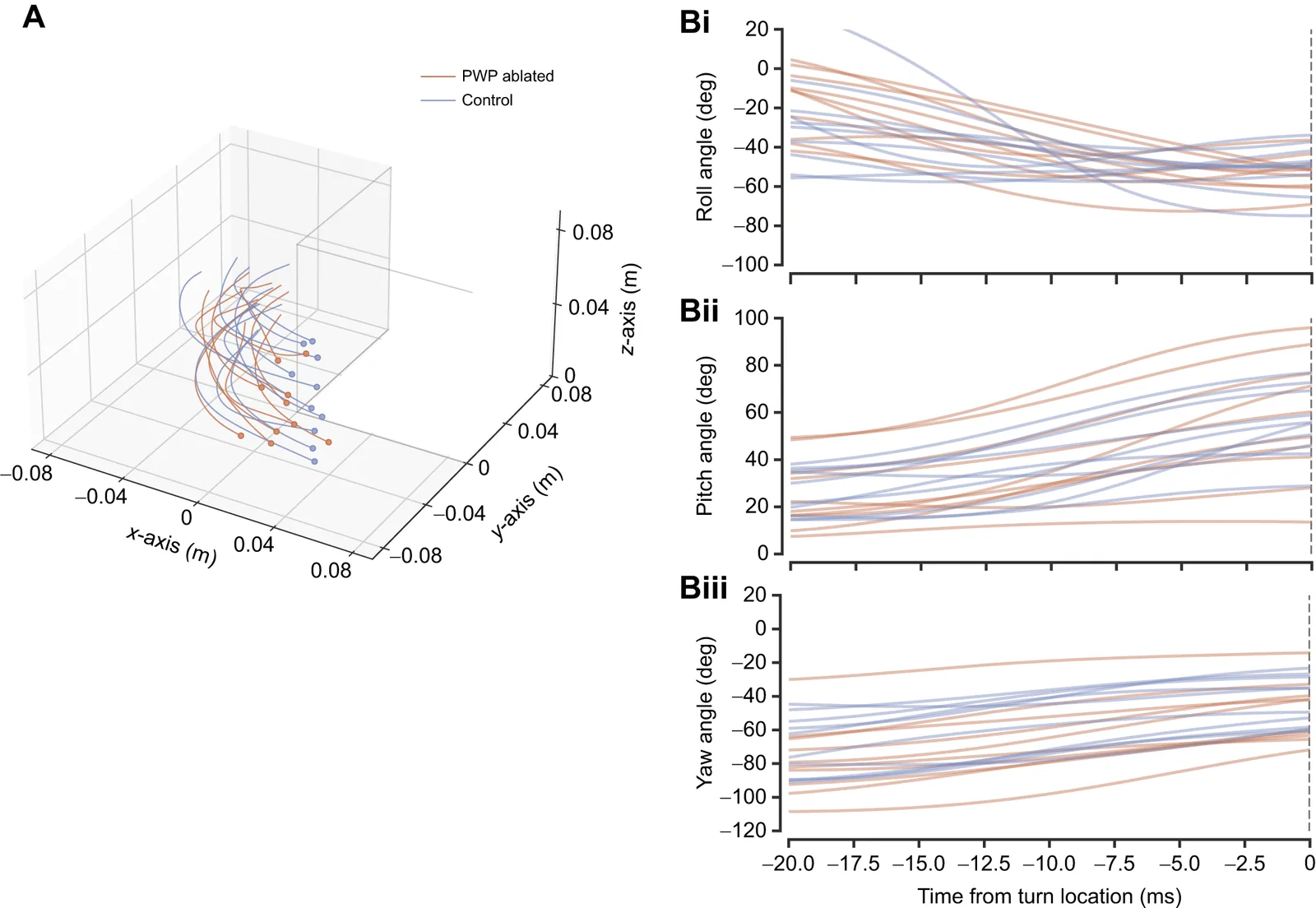
**Flight trajectories and yaw, pitch and roll dynamics during turning in control and PWP-ablated flies.** (A) Three-dimensional flight trajectories of all flies, plotted using the midpoint of the wing bases. (B) Roll (i), pitch (ii) and yaw (iii) traces aligned to the onset of the turn location, defined for each trial as the centripetal acceleration peak associated with the earliest prominent heading change during the maneuver. Traces are shown relative to this turn location, set to 0 ms.

### Whole-body kinematic parameters of control and PWP-ablated flies are comparable

We next analyzed flight speed and trajectory curvature to compare flight performance between control and PWP-ablated groups (a representative control trajectory is shown in Fig. 4A). The median of the trial-wise maximum speed in PWP-ablated flies was 0.96 m s^−1^, comparable to that of control flies (1.05 m s^−1^; Fig. 4B; *n*=10 per group, Mann–Whitney *U*-test, *P*=0.345; Table S1). Similarly, the median of the trial-wise median speed during the yaw maneuver was 0.99 m s^−1^ in controls and 0.95 m s^−1^ in PWP-ablated flies (Fig. 4C; *n*=10 per group, Mann–Whitney *U*-test, *P*=0.473; Table S1).

**Fig. 4.**
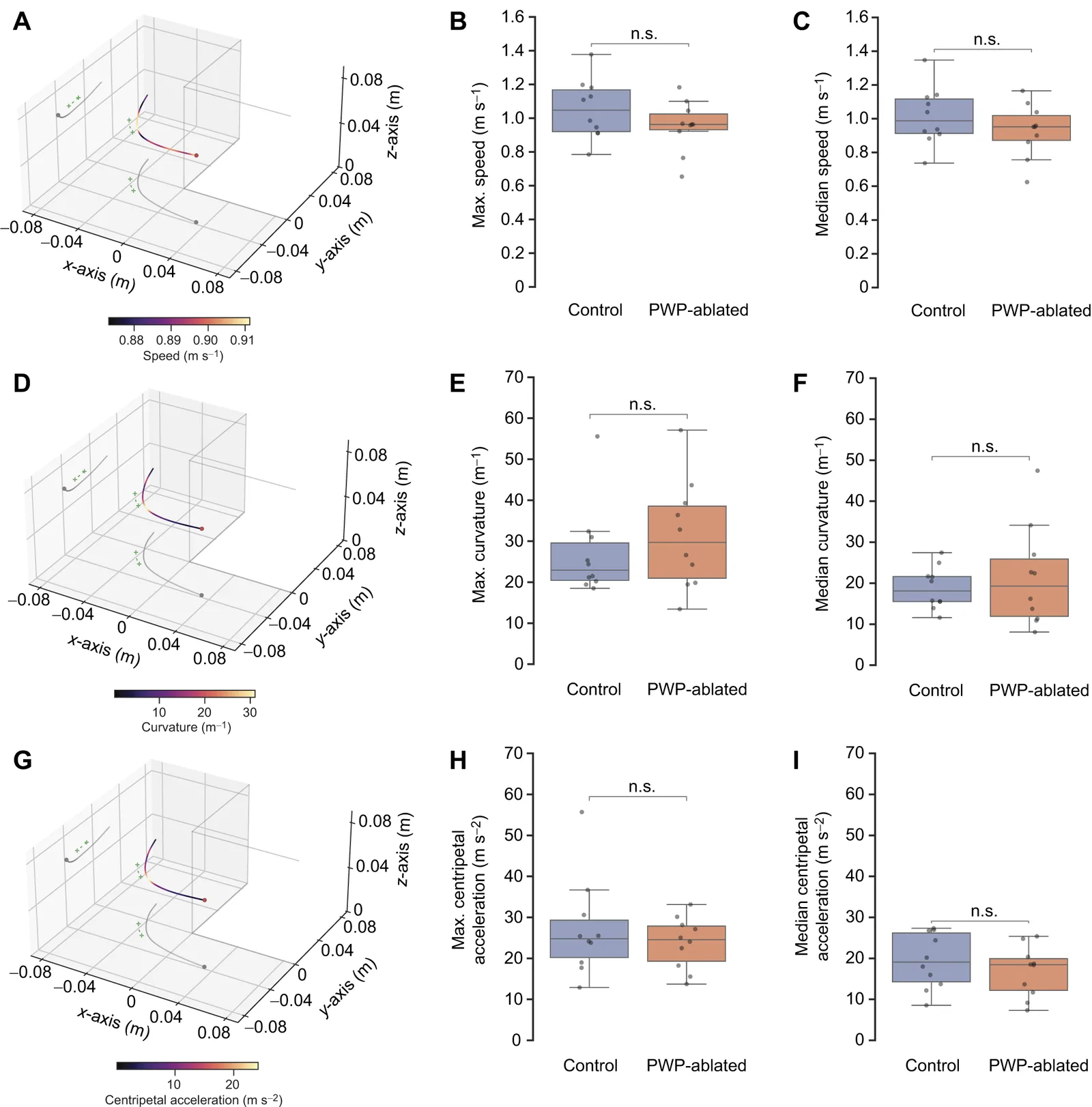
**Comparison of the whole-body kinematic parameters of control and PWP-ablated flies during the left yaw maneuver.** (A) A representative control fly trajectory, color coded by flight speed. (B,C) Trial-wise maximum (B) and median (C) flight speed during the maneuver. (D) A representative control fly trajectory, color coded by curvature. (E,F) Trial-wise maximum (E) and median (F) curvature. (G) A representative control fly trajectory, color coded by centripetal acceleration. (H,I) Trial-wise maximum (H) and median (I) centripetal acceleration. Group comparisons were performed using two-sided Mann–Whitney *U*-tests for all whole-body kinematic parameters (*n*=10 per group). n.s., not significant (*P*>0.05).

We then compared trajectory curvature in control and PWP-ablated groups (Fig. 4D). The median of the trial-wise maximum curvature in the PWP-ablated group was 29.72 m^−1^, comparable to that of control flies (22.94 m^−1^; Fig. 4E; *n*=10 per group, Mann–Whitney *U*-test, *P*=0.385; Table S1). Similarly, the median of the trial-wise median curvature was 18.12 m^−1^ in controls and 19.30 m^−1^ in PWP-ablated flies (Fig. 4F; *n*=10 per group, Mann–Whitney *U*-test, *P*=0.970; Table S1).

We next analyzed centripetal acceleration, defined as the product of curvature and the square of linear velocity (κ**v**^2^; Fig. 4G), which also did not differ between the two groups. The median of the trial-wise maximum centripetal acceleration in PWP-ablated flies was 24.55 m s^−2^, comparable to that of control flies (24.78 m s^−2^; Fig. 4H; *n*=10 per group, Mann–Whitney *U*-test, *P*=0.734; Table S1). Similarly, the median of the trial-wise median centripetal acceleration was 19.08 m s^−2^ in controls and 18.44 m s^−2^ in PWP-ablated flies (Fig. 4I; *n*=10 per group, Mann–Whitney *U*-test, *P*=0.427; Table S1).

Together, these results indicate that PWP ablation did not significantly alter flight performance as measured by speed, curvature or centripetal acceleration.

### Roll, pitch and yaw body angles during turning maneuvers are similar in control and PWP-ablated flies

To assess differences in flight dynamics between control and PWP-ablated flies, we compared the rates of change of pitch (Fig. 5A–C), yaw (Fig. 5D–F) and roll (Fig. 5G–I). These analyses revealed no significant differences between the two groups.

**Fig. 5.**
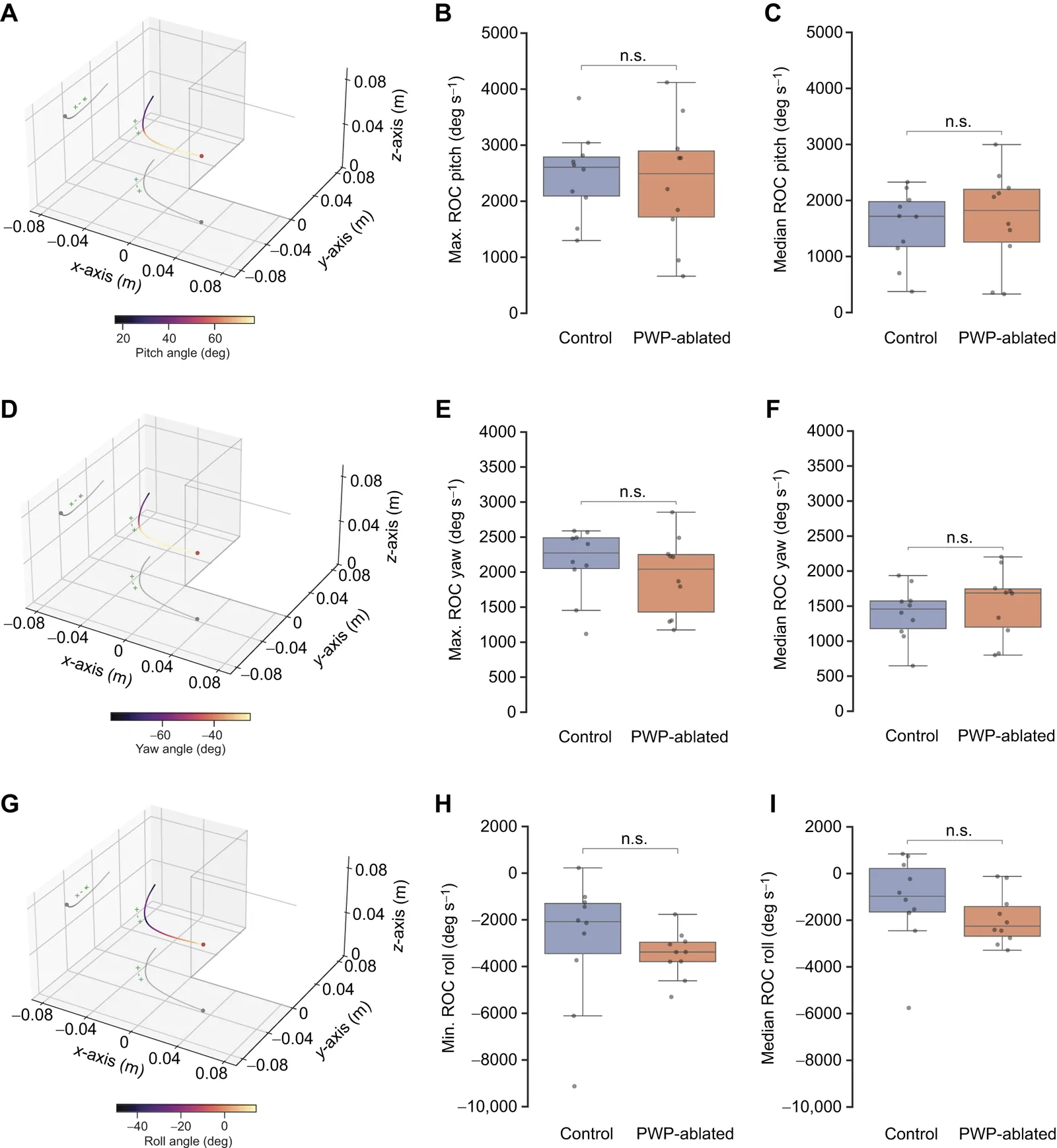
**Analysis of body angular velocity or rate of change (ROC) of pitch, yaw and roll during left yaw turning maneuvers in control and PWP-ablated flies.** (A) A representative flight trajectory, color coded by pitch angle. (B,C) Trial-wise maximum (B) and median (C) rates of change of pitch. (D) A representative flight trajectory, color coded by yaw angle. (E,F) Trial-wise maximum (E) and median (F) rates of change of yaw. (G) A representative flight trajectory, color coded by roll angle. (H,I) Trial-wise minimum (H) and median (I) rates of change of roll. Minimum roll rate was used because leftward turns were associated with negative roll values, so the minimum captured the largest leftward roll excursion. Group comparisons were performed using two-sided Mann–Whitney *U*-tests for pitch, yaw and roll (*n*=10 per group). n.s., not significant (*P*>0.05).

For pitch, the median of the trial-wise maximum pitch rate was 2605.82 deg s^−1^ in control flies and 2492.69 deg s^−1^ in PWP-ablated flies (Fig. 5B; *n*=10 per group, Mann–Whitney *U*-test, *P*=0.970; Table S1). Similarly, the median of the trial-wise median pitch rate was 1716.53 deg s^−1^ in controls and 1822.45 deg s^−1^ in PWP-ablated flies (Fig. 5C; *n*=10 per group, Mann–Whitney *U*-test, *P*=0.734; Table S1).

For yaw, the median of the trial-wise maximum yaw rate was 2273.04 deg s^−1^ in control flies and 2042.52 deg s^−1^ in PWP-ablated flies (Fig. 5E; *n*=10 per group, Mann–Whitney *U*-test, *P*=0.427; Table S1). Similarly, the median of the trial-wise median yaw rate was 1458.32 deg s^−1^ in controls and 1687.71 deg s^−1^ in PWP-ablated flies (Fig. 5F; *n*=10 per group, Mann–Whitney *U*-test, *P*=0.427; Table S1).

For roll, we quantified the minimum roll rate because leftward turning was represented by negative roll values, such that the minimum value captured the largest leftward roll excursion. The median of trial-wise minimum roll rate was −2076.96 deg s^−1^ in control flies and −3376.82 deg s^−1^ in PWP-ablated flies (Fig. 5H; *n*=10 per group, Mann–Whitney *U*-test, *P*=0.121; Table S1). One control fly showed a markedly greater minimum roll rate than the rest of the dataset, reaching −9127.93 deg s^−1^; however, the group comparison remained non-significant. Likewise, the median of the trial-wise median roll rate was −972.25 deg s^−1^ in controls and −2254.05 deg s^−1^ in PWP-ablated flies (Fig. 5I; *n*=10 per group, Mann–Whitney *U*-test, *P*=0.121; Table S1).

Together, these results indicate that PWP ablation did not significantly alter body angle dynamics during flight.

### PWP-ablated flies can modulate wingbeat amplitude

We next compared wingbeat frequency (Fig. 6A,B), wingbeat amplitude (Fig. 6C,D), wingbeat amplitude difference between the left and right wings (Fig. 6E,F), stroke plane angle (Fig. 6G,H) and stroke deviation (Fig. 6I,J) between control and PWP-ablated groups. The median of the trial-wise maximum wingbeat frequency was 285.7 Hz in both control and PWP-ablated flies (Fig. 6A; *n*=10 per group, Mann–Whitney *U*-test, *P*=0.937; Table S1). The median of the trial-wise median wingbeat frequency during the turn was 266.7 Hz in controls and 276.2 Hz in PWP-ablated flies (Fig. 6B; *n*=10 per group, Mann–Whitney *U*-test, *P*=0.457; Table S1).

**Fig. 6.**
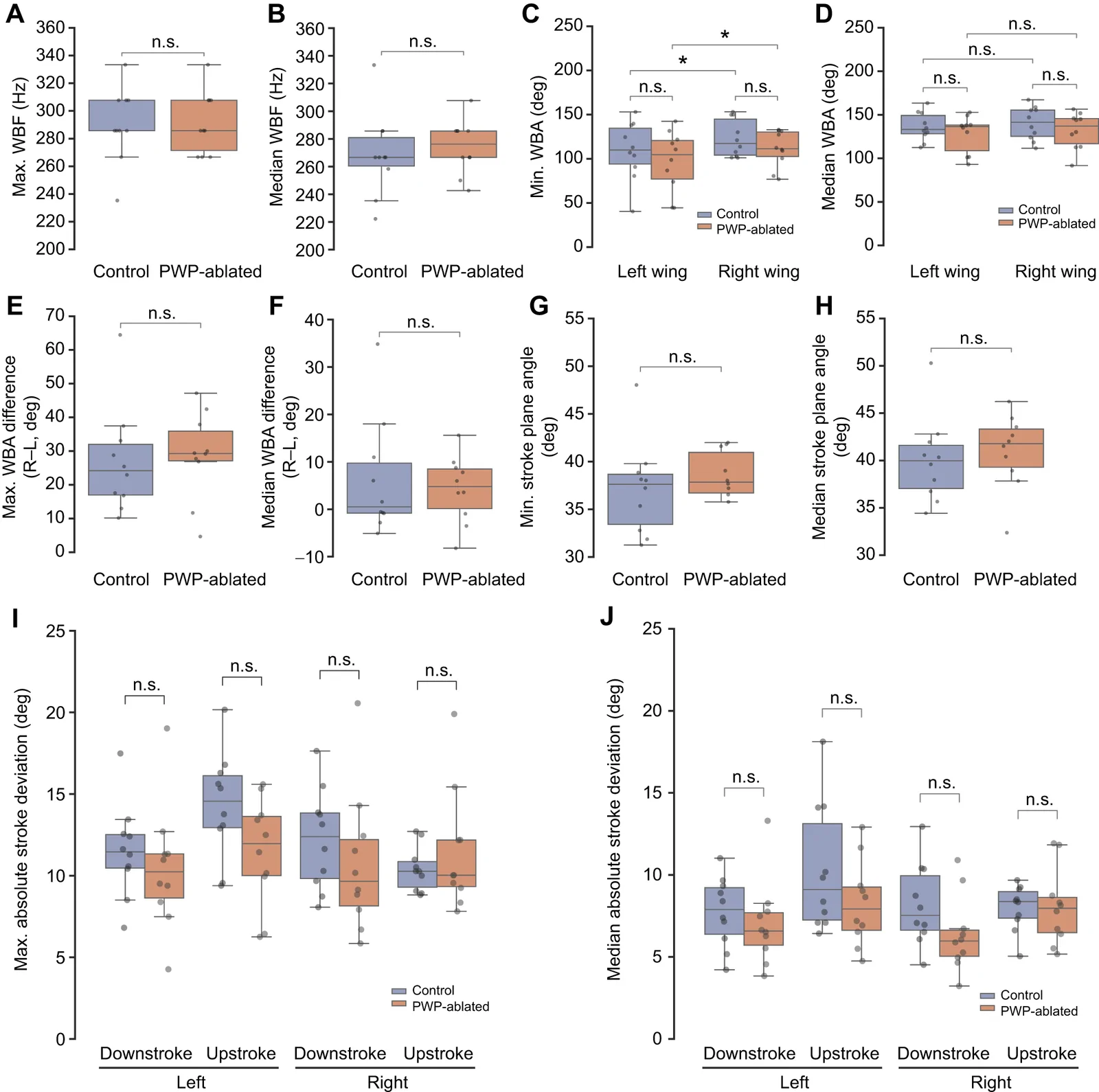
**Comparison of wingbeat frequency and amplitude between control and PWP-ablated flies during leftward yaw turn maneuvers.** (A,B) Maximum (A) and median (B) wingbeat frequency (WBF). (C,D) Minimum (C) and median (D) wingbeat amplitude (WBA) for left and right wings in control and PWP-ablated flies. (E,F) Maximum (E) and median (F) wingbeat amplitude difference (right minus left wingbeat amplitude). (G,H) Minimum (G) and median (H) stroke plane angle. (I,J) Maximum (I) and median (J) absolute stroke deviation angle across stroke cycles of left and right wings in control and PWP-ablated flies. Group comparisons were performed using two-sided Mann–Whitney *U*-tests, and within-group left–right comparisons for wingbeat amplitude were performed using paired Wilcoxon signed-rank tests (*n*=10 per group). n.s., not significant (*P*>0.05).

When comparing trial-wise minimum wingbeat amplitude during left turns, the left wing showed significantly lower values than the right wing in both control and PWP-ablated flies (Fig. 6C; *n*=10 per group, paired Wilcoxon signed-rank test, control *P*=0.020, PWP ablated *P*=0.049; Table S2), consistent with reduced left-wing amplitude during leftward turning. In control flies, the median of the trial-wise minimum wingbeat amplitude was 110.0 deg in the left wing and 117.3 deg in the right wing, whereas in PWP-ablated flies, the corresponding values were 104.6 deg and 111.4 deg. However, the minimum wingbeat amplitude of the left wing did not differ between control and PWP-ablated flies, and the same was true for the right wing (Fig. 6C; *n*=10 per group, Mann–Whitney *U*-test, left wing *P*=0.521, right wing *P*=0.427; Table S1).

When comparing trial-wise median wingbeat amplitude, no significant difference between the left and right wings was detected in either group. In control flies, the median of the trial-wise median wingbeat amplitude was 133.2 deg in the left wing and 141.5 deg in the right wing, whereas in PWP-ablated flies, the corresponding values were 136.5 deg and 137.1 deg (Fig. 6D; *n*=10 per group, paired Wilcoxon signed-rank test, control *P*=1.000, PWP ablated *P*=0.160; Table S2). The median amplitude of the left wing did not differ between control and PWP-ablated flies, and the same was true for the right wing (Fig. 6D; *n*=10 per group, Mann–Whitney *U*-test, left wing *P*=0.623, right wing *P*=0.385; Table S1).

For wingbeat amplitude difference, the median of the trial-wise maximum wingbeat amplitude difference was 24.2 deg in controls and 29.3 deg in PWP-ablated flies (Fig. 6E; *n*=10 per group, Mann–Whitney *U*-test, *P*=0.427; Table S1). Similarly, the median of the trial-wise median wingbeat amplitude difference was 0.5 deg in control flies and 4.8 deg in PWP-ablated flies (Fig. 6F; *n*=10 per group, Mann–Whitney *U*-test, *P*=1.000; Table S1).

For stroke plane angle, the median of the trial-wise minimum stroke plane angle was 37.6 deg in control flies and 37.8 deg in PWP-ablated flies (Fig. 6G; *n*=10 per group, Mann–Whitney *U*-test, *P*=0.623; Table S1). Similarly, the median of the trial-wise median stroke plane angle was 40.0 deg in controls and 41.8 deg in PWP-ablated flies (Fig. 6H; *n*=10 per group, Mann–Whitney *U*-test, *P*=0.345; Table S1).

We next compared stroke deviation between control and PWP-ablated flies by quantifying maximum absolute stroke deviation separately for the left and right wings during downstroke and upstroke. For the trial-wise maximum absolute stroke deviation (Fig. 6I), no significant differences were detected between groups for any segment. The median values were 11.5 deg in controls and 10.2 deg in PWP-ablated flies for the left downstroke (*P*=0.385; Table S1), 14.6 deg and 12.0 deg, respectively, for the left upstroke (*P*=0.089; Table S1), 12.4 deg and 9.7 deg, respectively, for the right downstroke (*P*=0.241; Table S1), and 10.3 deg and 10.0 deg, respectively, for the right upstroke (*P*=1.000; *n*=10 per group; Mann–Whitney *U*-test; Table S1).

A similar pattern was observed when comparing the median of the segment-wise maximum absolute stroke deviation (Fig. 6J). No significant group differences were detected for the left downstroke, left upstroke, right downstroke or right upstroke. The median values were 7.9 deg in controls and 6.6 deg in PWP-ablated flies for the left downstroke (*P*=0.345; Table S1), 9.1 deg and 7.9 deg, respectively, for the left upstroke (*P*=0.241; Table S1), 7.5 deg and 6.0 deg, respectively, for the right downstroke (*P*=0.076; Table S1), and 8.4 deg and 8.0 deg, respectively, for the right upstroke (*P*=0.678; *n*=10 per group; Mann–Whitney *U*-test; Table S1).

Together, these results show that flies turn by selectively reducing the minimum wingbeat amplitude of the inner left wing, and that this mechanism remains functional even after PWP ablation. Minimum and median wingbeat amplitude did not differ significantly between control and PWP-ablated flies, and the same was true for wingbeat amplitude difference. Likewise, neither stroke plane angle nor stroke deviation metrics differed between groups. Overall, these results indicate that the wing kinematic asymmetries associated with turning are largely preserved despite PWP ablation.

## DISCUSSION

The experiments described above were designed to explicitly test the gearbox hypothesis, by determining whether the interaction between the RS and PWP is necessary for wing amplitude modulation during aerial maneuvers. Our comparative analysis of wing hinge morphology across 10 fly taxa revealed substantial variability, particularly in the grooved structure of the PWP and in the presence of pterale C, which has been proposed to function either as a mechanosensor or mechanical damping element at ventral stroke reversal. Pterale C was absent in three of the 10 taxa examined. We next assayed yaw behaviors, which require asymmetric wing amplitude modulation and are coupled with pitch and roll. Our results show that even when RS–PWP interactions were disrupted by ablating the PWP, flies executed maneuvers that were indistinguishable from those of individuals with intact wing hinges. These findings suggest that the gearbox hypothesis requires revision and highlight the importance of steering muscles in mediating wing amplitude modulation.

### How does a fly generate a yaw turn?

In *Drosophila*, asymmetric modulation of wing kinematics can generate yaw torque by producing unequal aerodynamic forces between the left and right wings (Fry et al., 2003). Yaw control is further refined through modulation of wing rotation during stroke reversals, which alters wing angle of attack and consequently the aerodynamic forces generated by each wing (Muijres et al., 2014). Rapid changes in heading can also involve coordinated banked maneuvers, in which flies generate pitch and roll torques through changes in multiple wing kinematic parameters. In *Drosophila hydei*, such visually elicited evasive maneuvers enable exceptionally rapid reorientation, reaching angular velocities of approximately 5300 deg s^−1^, compared with approximately 1000 deg s^−1^ during voluntary saccadic turns (Muijres et al., 2014). During these maneuvers, the body rolls in the direction of the turn, redirecting aerodynamic forces laterally and thereby facilitating changes in heading (Dickinson and Muijres, 2016).

In all the experiments described here, flies performed yaw turns to change their flight direction (e.g. Fig. 4). Because pitch and roll are coupled during banked yaw maneuvers, this behavior offered an ideal context to examine subtle impairments from PWP ablation. Both control and PWP-ablated flies executed turns using a banked maneuver. Because their ability to coordinate pitch, roll and yaw during flight was unimpaired by PWP ablation, we conclude that the interaction between the RS and PWP may not be critical for generating complex maneuvers. Alternatively, in the absence of RS–PWP interactions, flies may rely on steering muscle activity to generate the necessary kinematic changes.

### Role of the gearbox in dipteran flight

The gearbox mechanism has also been identified in other insect orders, where it exhibits considerable variation despite the ability of these insects to modulate wingbeat amplitude. In flies, the interaction between the RS and multi-grooved PWP is believed to facilitate unilateral amplitude modulation. As observed in *S. dux* and other flies (Deora et al., 2015), the RS transiently contacts one of the PWP grooves during the downstroke, serving as a stop that constrains wingbeat amplitude to one of five modes (Fig. 1A). Mode 0 corresponds to the disengaged state, where the wing remains decoupled from thoracic vibrations. In contrast, modes 1–3 involve wing clutch engagement, each associated with a specific wingbeat amplitude (Nalbach, 1989; Deora et al., 2015). In modes 1, 2 and 3, the RS contacts peaks 1, 2 and 3 of the PWP, respectively, whereas in mode 4, the RS moves anteriorly without contact. These data are consistent with Nalbach's (1989) tethered-flight observations which described three modes based on amplitude distributions.

The gear change mechanism would require precise actuation within a 10 μm range at wingbeat frequencies exceeding 100 Hz. Such control would in turn require rapid sensory feedback regarding wing hinge configuration on a stroke-to-stroke basis, but the source of this feedback remains unclear. As shown here, the number of PWP grooves varies substantially among fly species with flight frequencies above 100 Hz, yet these species maintain continuous amplitude modulation. Furthermore, our SEM analyses across Oestroidea species (*Calliphora* spp., *S. dux* and *M. domestica*) revealed that the RS itself possesses a distinct groove and interlocks with the peak of the PWP. Modes 1, 2 and 3 are separately captured in Fig. 2I,H,J, respectively, albeit in different Oestroidea species.

Together, the experiments described here explicitly test the role of the gearbox in wing amplitude modulation during aerial maneuvers. Insects adjust wingbeat amplitude to generate asymmetrical forces for steering or to counteract deviations from stable flight. If the PWP plays a critical role in wingbeat amplitude modulation, its bilateral ablation should affect flight performance in *M. domestica*. The assay described here measured repeatable maneuvers requiring wingbeat amplitude modulation. However, our data showed no significant differences in flight parameters between flies with intact gearboxes and those with ablated PWPs. Body trajectory, body angle and wing angle remained comparable between groups. Rates of yaw, pitch and roll change were also similar, and wingbeat amplitude during turns did not differ.

Within the constraints of the experimental design, we may therefore conclude that the PWP is not necessary for wingbeat amplitude modulation and steering in flies. These conclusions challenge models that assign a central role to the PWP as a discrete ‘gearbox’ mechanism for dipteran amplitude modulation. An alternative hypothesis proposes that instead of functioning as gears, PWP grooves provide a textured surface that prevents the radial stop from slipping (Deora et al., 2017). In certain fly species, specific grooves may enhance friction, further reducing slippage between the RS and PWP. This perspective implies that modes 1 and 2 are not distinct states; rather, amplitude modulation within these modes results from differential activity in the underlying steering muscles. Consequently, the gearbox mechanism may be less discrete and more dynamic than previously assumed, as a result of the graded, combinatorial action of steering muscles.

### Role of steering muscles in amplitude modulation

Based on the experiments described above, we infer that the gearbox plays, at most, a limited role in regulating wingbeat amplitude, and propose that amplitude modulation is mediated primarily by steering muscles acting at the wing base. Although the contribution of steering muscles to wingbeat modulation has been investigated extensively (e.g. Nachtigall and Wilson, 1967; Wisser and Nachtigall, 1984; Tu and Dickinson, 1994, 1996; Trimarchi and Schneiderman, 1995; Fayyazuddin and Dickinson, 1996; Heide and Götz, 1996; Balint and Dickinson, 2001; Gordon and Dickinson, 2006; Walker et al., 2014; Sadaf et al., 2015; Lehmann and Bartussek, 2017; Lindsay et al., 2017; Melis et al., 2024), direct functional tests during flight have only recently become feasible, owing to the development of genetic tools in *D. melanogaster* that enable *in vivo* calcium imaging. In these studies, muscle activity is linked to wing hinge mechanics and wing kinematics to provide a systems-level understanding of how steering muscles control wing movement and flight maneuvers (Lindsay et al., 2017; Melis et al., 2024). By combining calcium recordings from approximately 12 steering muscles with high-speed 3D kinematic tracking, these studies showed that flight control emerges from coordinated activity across the muscle ensemble rather than from individual muscles. Steering muscles are organized into tonic muscles, which are constantly active and provide fine-scale stabilizing control, and phasic muscles, which are recruited transiently to generate rapid, high-amplitude maneuvers. These muscles are organized according to their wing hinge sclerites, forming modular motor units that influence specific aspects of wing motion.

These studies suggest that wing kinematic control is distributed across mechanical modules rather than mapped one-to-one between individual muscles and kinematic variables. By correlating muscle activity with detailed wing kinematics, these studies demonstrated that stereotyped activation patterns correspond to distinct flight maneuvers such as yaw, pitch and roll, often through bilaterally asymmetric activity. Thus, the wing hinge likely converts coordinated muscle inputs into complex, non-linear wing motions in a combinatorial manner. Muscle activity does not directly encode individual kinematic parameters; instead, wing motion emerges from the interaction between muscle forces and hinge mechanics. Melis et al. (2024) used *Drosophila* data to generate predictive, low-dimensional models that map muscle activity onto wing kinematics and body motion, indicating that a relatively small set of muscles can generate a wide range of controlled flight behaviors through structured, coordinated activation patterns. More broadly, coordinated motor program control has also been demonstrated in hawkmoth flight, where spike timing across muscles contributes to flight control (Ortega et al., 2019; Wood et al., 2024). These studies demonstrate the crucial role that coordinated muscle activity plays in determining wing kinematics.

### Role of pterale C in the wing hinge dynamics

Many aspects of individual gearbox components remain unexplored. For example, SEM of pterale C revealed a flattened, mushroom-like tip covered with fine bristle-like structures (Miyan and Ewing, 1984). Because the anterior wing margin contacts this structure at the end of each downstroke, previous researchers speculated that pterale C is stimulated at wingbeat frequency and may provide mechanosensory feedback on wing position or mechanically damp stroke transitions (Miyan and Ewing, 1984). Although extracellular recordings from the ventral base of pterale C indicated rhythmic activity correlated with wing motion (Miyan and Ewing, 1984), the origin of these signals was uncertain. Recent high-resolution connectomic reconstructions in *Drosophila* show that pterale C is not innervated by sensory neurons; rather, the radius, or radial vein, sensory nerve passes directly beneath it, suggesting that earlier electrophysiological recordings likely captured activity from this neighboring axon bundle (Lesser et al., 2026), effectively ruling out the hypothesis that pterale C functions as a proprioceptive organ. It remains possible that pterale C contributes passively to mechanical damping of the wing hinge. Because pterale C is absent in three of the 10 taxa surveyed here (Fig. 2A–J), it would be interesting to examine wing kinematics at ventral stroke reversal, where pterale C is hypothesized to serve as a damping element.

### Conclusions

The experiments presented here show that the proposed gearbox function of RS, PWP and pterale C needs to be revised. First, the geometry of these structures is highly variable even within dipteran taxa, which suggests that the structural features of the wing hinge gearbox are not highly conserved. Second, the ablation of the PWP does not substantially alter the flies' yaw turn performance, which is coupled to pitch and roll. Thus, the RS–PWP interaction is not necessary for the flies' ability to perform these turns. Indeed, neither their body kinematics nor their wing kinematics is significantly affected by PWP ablation, although we do not rule out that the RS–PWP interaction may serve to enhance friction or reduce slippage. Based on these data, we propose that amplitude modulation is primarily actuated by the steering muscles through their fine-tuned configuration of the wing hinge. Our studies also underscore the importance of direct testing of functional hypotheses, rather than their indirect inference from investigations at the systems level.

## Supplementary Material

10.1242/jexbio.250768_sup1 Supplementary information
