# Supplementary material for: Testing the gearbox hypothesis for insect flight control
Source: J Exp Biol. 2026 Oct 16;229(20):jeb250768. doi: 10.1242/jeb.250768 (PMC13723389; doi:10.1242/jeb.250768)
Supplement: Supplementary file 1 [file jexbio-229-250768-s1.pdf]

Table S1. Inter-group comparisons of flight and wing kinematics.

Control and PWP-ablated flies were compared using two-sided Mann–Whitney U tests. The table reports trial-wise summary metrics for whole-body kinematics, body-angle rates, wingbeat frequency, wingbeat-amplitude difference, wing-specific wingbeat amplitude, stroke-plane angle, and stroke-deviation measures, together with group sample sizes, test statistics, and p-values (n = 10 per group).

Flight parameter	Comparison	Control n	PWP ablated n	Test	U statistic	p value
Maximum curvature	Control vs PWP ablated	10	10	Mann-Whitney U	38	0.384673
Median curvature	Control vs PWP ablated	10	10	Mann-Whitney U	49	0.96985
Maximum centripetal acceleration	Control vs PWP ablated	10	10	Mann-Whitney U	55	0.73373
Median centripetal acceleration	Control vs PWP ablated	10	10	Mann-Whitney U	61	0.427355
Maximum speed	Control vs PWP ablated	10	10	Mann-Whitney U	63	0.344704
Median speed	Control vs PWP ablated	10	10	Mann-Whitney U	60	0.472676
Maximum yaw rate	Control vs PWP ablated	10	10	Mann-Whitney U	61	0.427355
Median yaw rate	Control vs PWP ablated	10	10	Mann-Whitney U	39	0.427355
Maximum pitch rate	Control vs PWP ablated	10	10	Mann-Whitney U	51	0.96985

Median pitch rate	Control vs PWP ablated	10	10	Mann-Whitney U	45	0.73373
Minimum roll rate	Control vs PWP ablated	10	10	Mann-Whitney U	71	0.121225
Median roll rate	Control vs PWP ablated	10	10	Mann-Whitney U	71	0.121225
Maximum wingbeat frequency	Control vs PWP ablated	10	10	Mann-Whitney U	51.5	0.937295
Median wingbeat frequency	Control vs PWP ablated	10	10	Mann-Whitney U	40	0.456854
Minimum wingbeat frequency	Control vs PWP ablated	10	10	Mann-Whitney U	59	0.508317
Wingbeat frequency standard deviation	Control vs PWP ablated	10	10	Mann-Whitney U	37	0.344523
Median stroke-plane angle	Control vs PWP ablated	10	10	Mann-Whitney U	37	0.344704
Maximum stroke-plane angle	Control vs PWP ablated	10	10	Mann-Whitney U	35	0.273036
Minimum stroke-plane angle	Control vs PWP ablated	10	10	Mann-Whitney U	43	0.623176
Median wingbeat-amplitude difference	Control vs PWP ablated	10	10	Mann-Whitney U	50	1
Maximum wingbeat-amplitude difference	Control vs PWP ablated	10	10	Mann-Whitney U	39	0.427355
Minimum wingbeat-amplitude difference	Control vs PWP ablated	10	10	Mann-Whitney U	32	0.185877

Journal of Experimental Biology • Supplementary information

Minimum wingbeat amplitude (left wing)	Control vs PWP ablated	10	10	Mann-Whitney U	59	0.520523
Minimum wingbeat amplitude (right wing)	Control vs PWP ablated	10	10	Mann-Whitney U	61	0.427355
Maximum wingbeat amplitude (left wing)	Control vs PWP ablated	10	10	Mann-Whitney U	63	0.344704
Maximum wingbeat amplitude (right wing)	Control vs PWP ablated	10	10	Mann-Whitney U	61	0.427355
Median wingbeat amplitude (left wing)	Control vs PWP ablated	10	10	Mann-Whitney U	57	0.623176
Median wingbeat amplitude (right wing)	Control vs PWP ablated	10	10	Mann-Whitney U	62	0.384673
Maximum absolute stroke deviation (left downstroke)	Control vs PWP ablated	10	10	Mann-Whitney U	62	0.384673
Maximum absolute stroke deviation (left upstroke)	Control vs PWP ablated	10	10	Mann-Whitney U	73	0.088973
Maximum absolute stroke deviation (right downstroke)	Control vs PWP ablated	10	10	Mann-Whitney U	66	0.241322
Maximum absolute stroke deviation (right upstroke)	Control vs PWP ablated	10	10	Mann-Whitney U	50	1

Journal of Experimental Biology • Supplementary information

Median maximum absolute stroke deviation (left downstroke)	Control vs PWP ablated	10	10	Mann-Whitney U	63	0.344704
Median maximum absolute stroke deviation (left upstroke)	Control vs PWP ablated	10	10	Mann-Whitney U	66	0.241322
Median maximum absolute stroke deviation (right downstroke)	Control vs PWP ablated	10	10	Mann-Whitney U	74	0.075662
Median maximum absolute stroke deviation (right upstroke)	Control vs PWP ablated	10	10	Mann-Whitney U	56	0.677585

Table S2. Intra-group paired comparisons of wingbeat amplitude.

Left and right wings were compared within each group using paired Wilcoxon signed-rank tests. The table reports trial-wise minimum, maximum, and median wingbeat amplitude for control and PWP-ablated flies, together with left-wing and right-wing medians, paired sample size, test statistic, and p-value (n = 10 per group).

Wing parameter	Group	Comparison	Paired n	Test	W statistic	p value
Minimum wingbeat amplitude	Control	Left wing vs right wing	10	Wilcoxon signed-rank	5	0.019531
Minimum wingbeat amplitude	PWP ablated	Left wing vs right wing	10	Wilcoxon signed-rank	8	0.048828
Maximum wingbeat amplitude	Control	Left wing vs right wing	10	Wilcoxon signed-rank	21	0.556641
Maximum wingbeat amplitude	PWP ablated	Left wing vs right wing	10	Wilcoxon signed-rank	21	0.556641
Median wingbeat amplitude	Control	Left wing vs right wing	10	Wilcoxon signed-rank	27	1
Median wingbeat amplitude	PWP ablated	Left wing vs right wing	10	Wilcoxon signed-rank	13	0.160156

Journal of Experimental Biology • Supplementary information

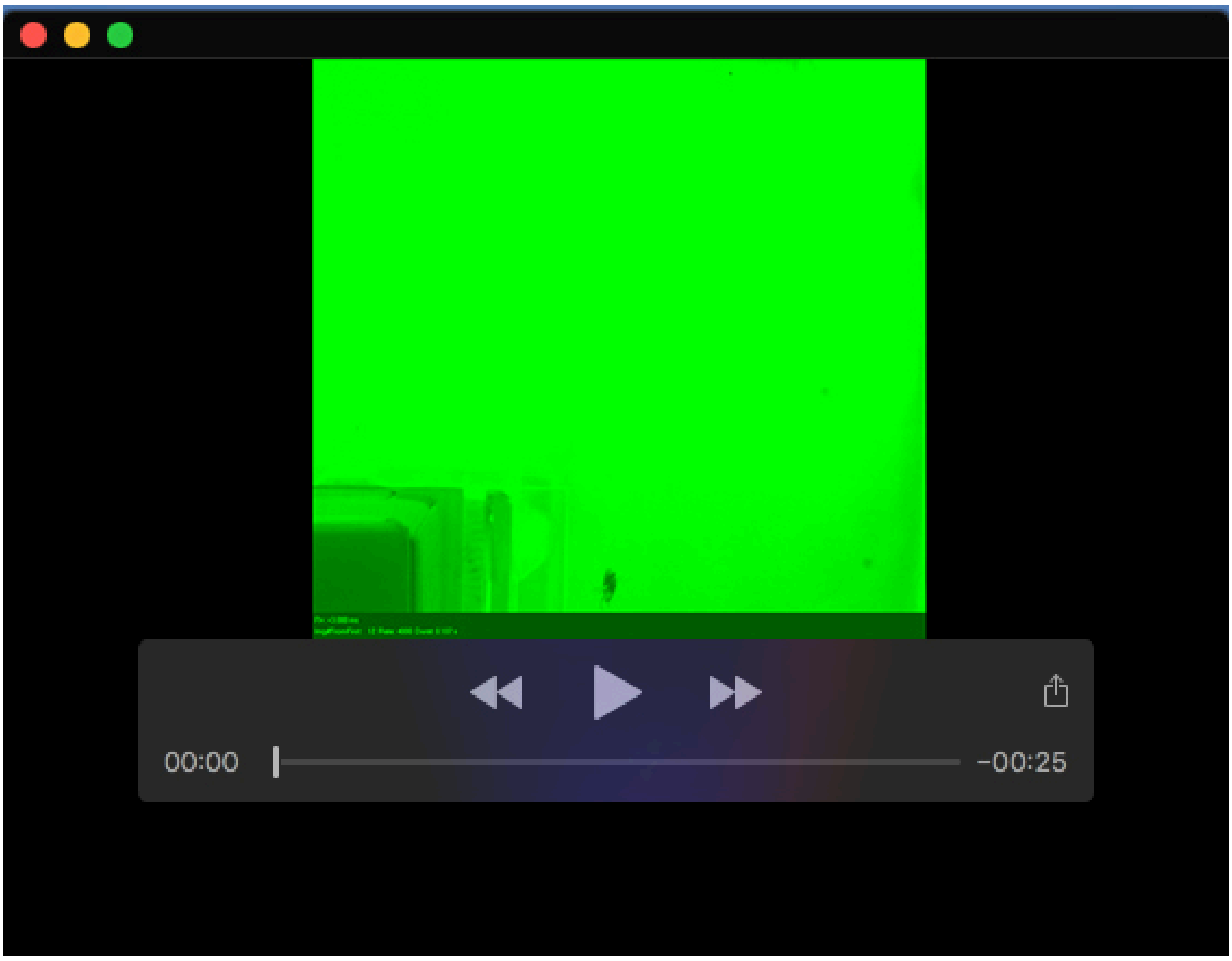

Movie 1. Top view of a *Musca domestica* performing a left yaw turn maneuver.

The video was acquired at 4000 frames per second and is played back at 30 frames per second. The fly approaches from arm A of the L-box, rolls leftward, pitches upward, and then counter-rolls, effectively executing a banked turn.

Journal of Experimental Biology • Supplementary information
